# Dynamic and redundant regulation of LRRK2 and LRRK1 expression

**DOI:** 10.1186/1471-2202-8-102

**Published:** 2007-11-28

**Authors:** Saskia Biskup, Darren J Moore, Alexis Rea, Bettina Lorenz-Deperieux, Candice E Coombes, Valina L Dawson, Ted M Dawson, Andrew B West

**Affiliations:** 1 Institute for Cell Engineering and Department of Neurology, Johns Hopkins University School of Medicine, Baltimore, USA; 2 Institute of Genetic Medicine, Johns Hopkins University School of Medicine, Baltimore, USA; 3 Institute of Human Genetics, GSF National Research Center for Environment and Health, Munich-Neuherberg, Germany; 4 Department of Molecular Biology and Genetics, Johns Hopkins University School of Medicine, Baltimore, USA; 5 Center for Neurodegeneration and Experimental Therapeutics and Department of Neurology, University of Alabama at Birmingham, Birmingham, USA

## Abstract

**Background:**

Mutations within the *leucine-rich repeat kinase 2 *(*LRRK2*) gene account for a significant proportion of autosomal-dominant and some late-onset sporadic Parkinson's disease. Elucidation of LRRK2 protein function in health and disease provides an opportunity for deciphering molecular pathways important in neurodegeneration. In mammals, LRRK1 and LRRK2 protein comprise a unique family encoding a GTPase domain that controls intrinsic kinase activity. The expression profiles of the murine LRRK proteins have not been fully described and insufficiently characterized antibodies have produced conflicting results in the literature.

**Results:**

Herein, we comprehensively evaluate twenty-one commercially available antibodies to the LRRK2 protein using mouse *LRRK2 *and human *LRRK2 *expression vectors, wild-type and *LRRK2*-null mouse brain lysates and human brain lysates. Eleven antibodies detect over-expressed human LRRK2 while four antibodies detect endogenous human LRRK2. In contrast, two antibodies recognize over-expressed mouse LRRK2 and one antibody detected endogenous mouse LRRK2. LRRK2 protein resides in both soluble and detergent soluble protein fractions. *LRRK2 *and the related *LRRK1 *genes encode low levels of expressed mRNA species corresponding to low levels of protein both during development and in adulthood with largely redundant expression profiles.

**Conclusion:**

Despite previously published results, commercially available antibodies generally fail to recognize endogenous mouse LRRK2 protein; however, several antibodies retain the ability to detect over-expressed mouse LRRK2 protein. Over half of the commercially available antibodies tested detect over-expressed human LRRK2 protein and some have sufficient specificity to detect endogenous LRRK2 in human brain. The mammalian LRRK proteins are developmentally regulated in several tissues and coordinated expression suggest possible redundancy in the function between *LRRK1 *and *LRRK2*.

## Background

Mutations in the *leucine-rich repeat kinase 2 *(*LRRK2*, OMIM 609007) gene are the most common known cause for sporadic and familial Parkinson's Disease (PD) [1-4]. The most frequent mutation in North America and Europe is a glycine to serine substitution at residue 2019 (G2019S) in the LRRK2 protein which accounts for up to 10% in familial cases and up to 2% in sporadic cases [5-9]. Frequencies of *LRRK2 *mutations in PD cases are reported as high as 40% in North Africans and Ashkenazi Jews [10,11], but since penetrance of the mutation in 80 year old carriers may be as low as 30% with standard diagnosis [12] the discrimination between familial and sporadic disease becomes difficult [13]. *LRRK2 *mutation carriers present with similar clinical and neurochemical features to sporadic disease, and with only few exceptions, present with typical Lewy body pathology [14,15].

Due to the unprecedented frequency of mutations in *LRRK2 *occurring in PD, a number of laboratories have embarked on the development of *LRRK2*-null and *LRRK2 *transgenic animals. In multiple-tissue Northern blots, LRRK2 is nearly constitutively expressed but the timing of expression has not been described nor the profile of the related *LRRK1 *gene [1,2,16]. Given the sequence similarity between *LRRK1 *and *LRRK2*, a complete expression profile may indicate whether redundancy in function could mask a phenotype in transgenic and knock-out animals. In addition, inappropriate expression in transgenic animals due to off-tissue expression or abnormal developmental expression may initiate undesirable phenotypes and reduce the utility as a PD model. To provide the complete expression profile of *LRRK2 *and *LRRK1*, we utilize quantitative PCR, Northern blot and Western blot experiments to track levels of expression.

The 2527 amino acid multi-domain protein kinase LRRK2 is associated with membranes in rodent brain and is present in tissues relevant to PD in human and rodent brain [17-20]. Some studies suggest the presence of LRRK2 protein in disease-associated aggregations such as Lewy bodies and Lewy neurites, using different antibodies against human LRRK2 [21-23]. To aid the neurodegeneration research community we present a detailed characterization of twenty-one commercially available antibodies to mouse and human LRRK2 purported as specific to the 280 kDa protein.

## Results

### LRRK2 is present in both freely soluble and SDS-soluble fractions of mouse brain lysate as determined by antibody JH5514

**Figure 1:**
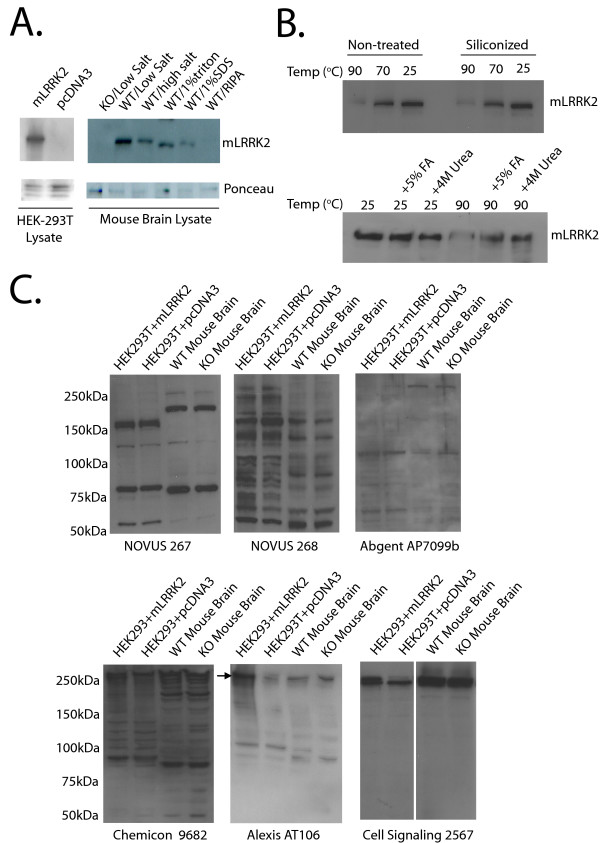
**Biochemical characterization of LRRK2 and testing of LRRK2 antibodies on mouse tissue**. A) Over-expressed mouse LRRK2 detected by JH5514 versus empty vector transfected HEK-293T cells. Whole mouse brain was mechanically homogenized in either PBS (low salt buffer), high salt buffer (PBS supplemented to 600 mM NaCl), 1% Triton X-100 (in PBS), 1% SDS (in PBS) or RIPA (1% SDS, 1% sodium deoxycholate, 1% NP-40). All buffers contained complete protease inhibitors (Roche). A knockout mouse brain is homogenized in PBS (low salt) alone and shown as control. B) Lysates derived from HEK-293T cells transfected with mLRRK2 plasmids were incubated in 1 × Laemmli sample buffer at the indicated temperatures for 10 minutes and then analyzed by SDS-PAGE. Plasticware used to process the protein samples were either siliconized or left untreated. Indicated samples were supplemented with 5% Formic acid (FA) or 4M urea after 10 minute incubation at the indicated temperature. C) LRRK2 antibodies Novus 267 and 268, Abgent AP7099b, and Chemicon (AB9682) in addition to Alexis (AT106) and Cell Signaling (2567) were tested on over-expressed mouse LRRK2 protein and wildtype and *LRRK2*-deficient mouse brain homogenized in PBS alone. All antibodies recognize cross-reactive bands near the expected size of LRRK2. An arrow denotes the position of LRRK2 in the Alexis (AT106) blot. All antibodies were tested on at least two independent membranes and lysates using optimized exposure conditions with similar results.

Published literature to date suggests many commercially available antibodies raised against peptides derived from human LRRK2 sequence detect a protein of approximately 280 kDa in size in mouse or human tissue and/or cell lines [21-28]. To confirm whether these antibodies detect mouse LRRK2 or cross-reactive protein species unrelated to LRRK2 migrating near 280 kDa, we performed immunoblot analyses of the various antibodies in wild type mice and LRRK2 knock-out mice in which exons 39 and 40 are disrupted (S. Biskup, M. Sasaski, V.L. Dawson and T.M. Dawson, personal observation) (Figure 1). Our previously characterized antibody JH5514 recognizes human over-expressed and endogenous LRRK2 protein in mouse and humans with minimal cross-reactivity with other protein species and LRRK2 immunoreactivity is absent in *LRRK2 *KO mice (Figure 1A). No endogenous LRRK2 can be detected in HEK-293T cells even with long exposures and high antibody concentrations (Figure 1A and data not shown). Our validated antibody JH5514 does not detect a protein of the appropriate size, determined through alignment with recombinant LRRK2 protein, in any tested cell line lysate including human embryonic kidney (HEK-293T) cells, human neuroblastoma (SH-SY5Y) cells, human neuroblastoma (BE(2)-M17) cells and rat adrenal (PC-12) cells (data not shown). Several of these cell lines, including HEK-293T and SH-SY5Y express a low level of LRRK2 mRNA compared with brain tissue suggesting post-transcriptional regulation (data not shown).

With a series of different extraction buffers for mouse brain using *LRRK2*-null brain tissue as a control, we show that membrane disruption through simple mechanical homogenization of brain tissue in PBS (low salt buffer) or in high salt buffer is sufficient to extract the majority of mouse LRRK2 that can be detected by Western Blot (Figure 1A). Mouse LRRK2 protein is also detected in relatively insoluble fractions only resolved through the addition of 1% SDS buffer. Incubation of lysate samples supplemented with 1× Laemmli sample buffer for ten minutes at temperatures above room temperature led to a loss of recoverable levels of LRRK2 (Figure 1B). This phenomenon is apparently not due to an interaction with plastic disposables since siliconized tubes and tips had no effect. Supplementation of the sample buffer lysate solution with either 4 M urea or 5% Formic acid (FA) after incubation for ten minutes at 90°C resulted in a significant recovery of LRRK2 protein levels, suggesting that LRRK2 may form SDS-resistant insoluble aggregates with elevated temperatures (Figure 1B).

### Commercially available antibodies that recognize over-expressed and endogenous mouse LRRK2

Using a panel of over-expressed mouse *LRRK2 *(*mLRRK2*) versus empty vector transfected HEK-293T cells in combination with *LRRK2*-null versus wild-type mouse brain tissue, we tested twenty-one commercially available LRRK2 antibodies to determine specificity. Most of the antibodies cannot detect over-expressed mLRRK2 or endogenous wild-type mLRRK2 protein (For a summary, see Table 1). For example, the antibodies Novus 267, Novus 268, Abgent AP7099b do not have the sufficient specificity to detect mLRRK2, either over-expressed or endogenous using standard blotting conditions (Figure 1C). Of note, antibody AP7099b from Abgent was raised against a peptide identical between mouse and human. Typical for the majority of antibodies tested here, cross-reactive species near the molecular weight of LRRK2 protein confound the interpretation of blots when appropriate controls such as *LRRK2*-null brain lysate are not included. With very high concentrations and exposure times, many cross reactive bands appear near the expected size of LRRK2 protein but none are changed in the *LRRK2*-null brain lysate for all commercially available antibodies with the exception of the Alexis (AT106) antibody, which recognizes a faint band immediately under a prominent cross-reactive protein species near 280 kDa (Figure 1C). Other antibodies capable of detecting over-expressed *mLRRK2 *in HEK 293T cells such as the Cell Signaling (2567) and Chemicon (ab9682) antibodies recognize cross-reactive protein species that presumably mask endogenous mLRRK2 protein due to the insufficient separation afforded by commercially available gradient mini-gels.

**Table 1:** Summary of antibodies tested on Western Blot

**Antibody**	**Overexpressed mLRRK2**	**Overexpressed hLRRK2**	**Endogenous mLRRK2**	**Endogenous hLRRK2**	**Peptide Location**
Abgent AP7099a	-	-	-	-	N/A
Abgent AP7099b	-	-	-	-	(LRR, AA 1246–1265, 100% identity) H: WSRVEKLHLSHNKLKEIPPE M: WSRVEKLHLSHNKLKEIPPE
Abgent AP7099c	-	-	-	-	(N-Term, around **L229**) H: EEIVLHVLHCLHSLAIPCNNVEVLMSGNVR M: KEIVYHVLCCLHS**L**AVTCSNVEVLMSGNVR
Abgent AP7099d	-	-	-	-	(N-Term, around **E285**) H: VSCCLLHRLTLGNFFNILVLNEVHEFVVKA M: VSCSLFQKLTLGNFFNILVLNEVHVFVVKA
Abgent AP7099e	-	+	-	+	(N-Term, around **E519**) H: RAILHFIVPGMPEESREDTEFHHKLNMVKK M: RAILHFVVPGLLEESRE..DSQCRPNVLRK
Abgent AP7099f	-	+	-	-	(N-Term, around **L893**) H: AQSDDLDSEGSEGSFLVKKKSNSISVGEFY M: GQSDDLDSEGSESSFLVKRKSNSISVGEVY
Abgent AP7099g	-	+	-	-	(N-Term, around **L899**) H: AQSDDLDSEGSEGSFLVKKKSNSISVGEFY M: GQSDDLDSEGSESSFLVKRKSNSISVGEVY
Abgent AP7099h	-	+	-	-	(N-Term, around **L955**) H: IFDHEDLLKRKRKILSSDDSLRSSKLQSHM M: VFDHEDLLRRKRKILSSDESLRSSRLPSHM
Abgent AP7099i	-	-	-	-	(C-Term, around **C2354**) H: STERNVMWGGCGTKIFSFSNDFTIQKLIET M: SSERHITWGGCGTKVFSFSNDFTIQKLIET
Abgent AP7099j	-	-	-	-	(C-Term, around **K2402**) H: CVHFLREVMVKENKESKHKMSYSGRVKTLC M: CVHFLKEVMVKLNKESKHQLSYSGRVKALC
Abgent AP7099k	-	-	-	-	(C-Term, around **Y2475**) H: MTAQLGSLKNVMLVLGYNRKNTEGTQKQKE M: ATAQLGSLKNVMLVLGYKRKSTEGIQEQKE
Abgent AM7099a	-	-	-	-	(monoclonal) N/A
Abgent AM7099b	-	-	-	-	(monoclonal) N/A
Abcam 27482	-	+	-	-	N/A
Abcam 19906	-	-	-	-	N/A
Novus Biological 267	-	+	-	+	(N-Term, AA 920–945) H: SNSISVGEFYRDAVLQRCSPNLQRHS M: SNSISVGEVYRDLALQRYSPNAQRHS
Novus Biological 268	-	+	-	+	(C-Term, AA 2500–2527) H: INLPHEVQNLEKHIEVRKELAEKMRRTSVE M: LNLPHEVQNLEKHIEVRTELADKMRKTSVE
Chemicon AB9682	-	+	-	-	N/A, synthetic peptide from rat
Chemicon AB9704	-	+	-	-	N/A
Alexis (AT106)	+	+	+	+	(AA 1838–2133)
Cell Signaling 2567	+	+	-	-	(C-Term, G2090) H: FPNEFDELEIQGKLPDPVKEYGCAPWPMVE M: FPNEFDELAIQGKLPDPVKEYGCAPWPMVE
JH5514	+	+	+	+	(C-Term, AA 2500–2515), 100% identity H: INLPHEVQNLEKHIE M: LNLPHEVQNLEKHIE

Summary of the results of commercially available antibodies against LRRK2 tested on Western Blot. A (+) denotes antibodies that produce a discernable band specific to LRRK2 protein. A (-) denotes antibodies unable to detect a band specific to LRRK2 protein. Antibodies were designed against the human sequence unless otherwise indicated.

### Commercially available antibodies that recognize over-expressed and endogenous human LRRK2

**Figure 2:**
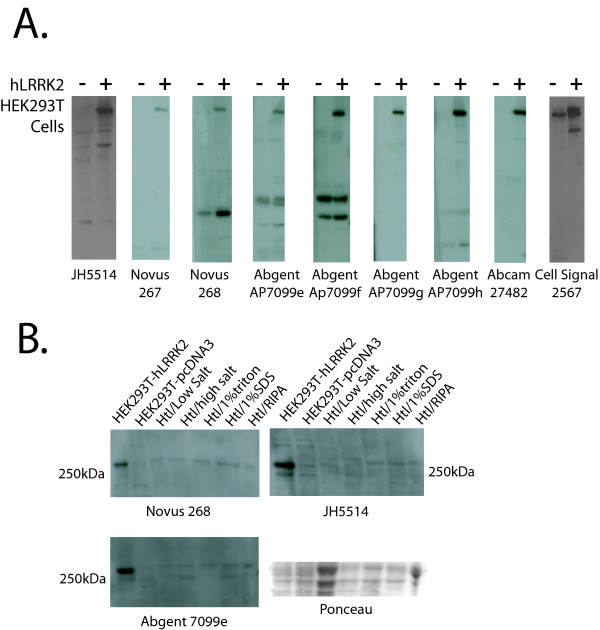
**Testing of LRRK2 antibodies on human tissue**. A) Antibodies able to detect over-expressed human LRRK2 protein. B) Fresh frozen tissue samples from the human temporal lobe (Htl) is subjected to different extraction buffers as in figure legend 1A. Novus 267, Novus 268, Abgent AP7099e and JH5514 have sufficient specificity to detect endogenous human LRRK2. All antibodies were tested on at least two independent membranes and lysates using optimized exposure conditions with similar results.

All antibodies were additionally tested on human over-expressed LRRK2 versus empty vector transfected HEK-293T cells (Table 1). Figure 2A shows the panel of antibodies capable of detecting over-expressed human LRRK2 (Novus 267, Novus 268, Abgent AP7099e, AP7099f, AP7099g, AP7099h, Abcam 27482 and Cell Signaling 2567). We tested antibodies capable of robustly detecting over-expressed human LRRK2 protein (Novus 267, Novus 268, Abgent AP7099e, AP7099f, AP7099g, AP7099h, Abcam 27482) using human brain lysate derived from fresh-frozen samples of human temporal lobe. *In situ *hybridization analyses have previously demonstrated *LRRK2 *mRNA is at the highest levels in the cortex and striatum and we would expect significant quantities of LRRK2 protein in the temporal lobe [16,18,29-32]. Novus 267, Novus 268 and AP7099e all have sufficient specificity to detect human LRRK2 based on their ability to resolve a band of the predicted molecular weight of LRRK2 with identical solubility profiles (Figure 2B). Novus 268 and AP7099e are shown in comparison with our in-house antibody JH5514 (Figure 2B). Using gel systems other than Tris-Glycine such as Tris-Acetate or Bis-Tris gels causes a dramatically different migration of LRRK2 protein with respect to protein markers, with migration near 240 kDa in Tris-Acetate gels (data not shown).

We visualized LRRK2 protein by Western blot in human brain lysates derived from tissue with minimal delay in processing (fresh frozen brain samples) and have been unsuccessful in detecting LRRK2 protein from the vast majority of archived frozen brain samples that correspond to typical post-mortem delays (4–24 hours) (data not shown). This may be due to a high rate of degradation or processes that otherwise render LRRK2 inaccessible to detection by immunoblotting due to typical post-mortem delay in most human tissue samples available. We have not observed significant decay in LRRK2 protein within protein lysate when stored at -20 or -80 degrees or dramatic loss of LRRK2 due to freeze/thaw cycles (data not shown).

### LRRK1 and LRRK2 expression in development and adulthood

**Figure 3:**
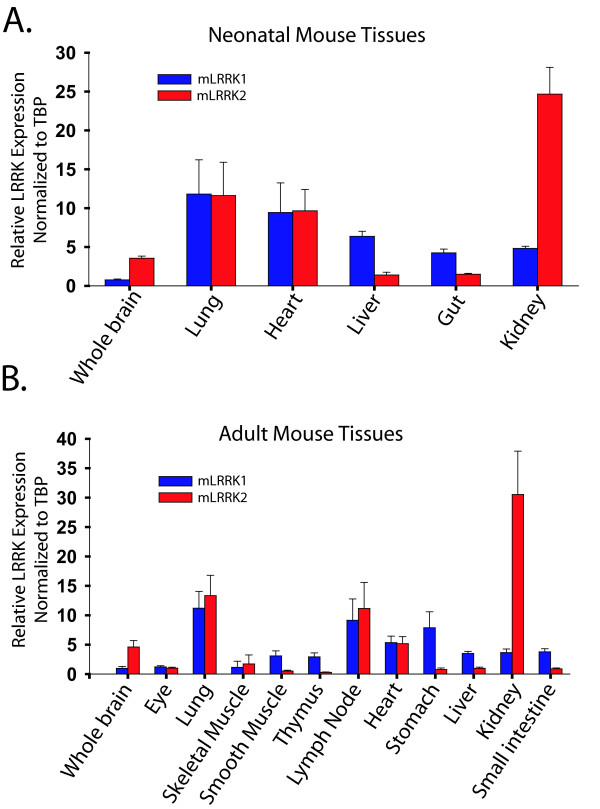
**LRRK1 and LRRK2 mRNA levels in development and adulthood**. A) Neonatal *LRRK1 *and *LRRK2 *mRNA levels. Relative *LRRK *expression was calculated by normalization to *TBP *(*TATA binding protein*) within each tissue sample using the delta delta CT method. Similar results were obtained with internal normalization to *GAPDH*. Error bars represent standard error mean derived from two each independently analyzed male and female 14 day old CD-1 mice B) *LRRK1 *and *LRRK2 *mRNA levels in adult mouse tissues as normalized to *TBP*. Similar results were obtained with normalization to *GAPDH*. Error bars represent standard error mean independently derived from two each female and male 3 month old CD-1 mice.

Many invertebrate organisms such as Drosophila and lower vertebrate organisms such as Fugu and Zebrafish possess only a single *LRRK *gene, suggesting mammalian LRRK1 and LRRK2 protein diverged from a single founder [33]. *LRRK*-like proteins and orthologues extend well beyond complex invertebrates into even simple single-celled eukaryotic organisms [34], suggesting a fundamental albeit unknown role for the *LRRK *family of proteins in mammals. Northern blot analysis has previously shown a broad distribution of *LRRK2 *mRNA in the brain and other organs in humans [1,2]. In order to better understand the regulation of *LRRK2 *expression we measured *LRRK1 *and *LRRK2 *mRNA in multiple organs in neonatal and adult mice (Figure 3A, B). We observed statistically identical levels of *LRRK1 *and *LRRK2 *mRNA in the lung, heart, skeletal muscle and lymph node (Figure 3B). *LRRK2 *mRNA is more abundant in kidney and brain tissue, whereas *LRRK1 *mRNA is more abundant in the gut.

**Figure 4:**
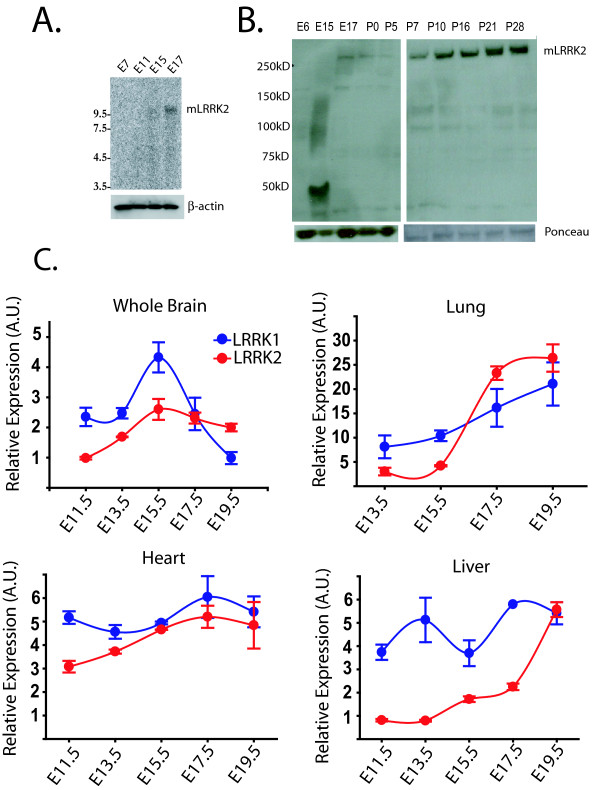
**LRRK1 and LRRK2 mRNA levels in development and adulthood**. A) Northern blot analysis of different embryonic stages (whole mouse embryo) using a *LRRK2 *specific riboprobe. B) LRRK2 protein can be detected by JH5514 in whole brain lysates on E17 and throughout post-natal development C) Relative *LRRK1 *and *LRRK2 *mRNA levels in direct comparison calculated from whole brain, lung, heart and liver during embryonic stages E11.5 to E19.5, with internal normalization to *TBP *transcript. Error bars represent standard error mean independently derived from a total of four CD-1 embryos at the indicated age.

Using Northern (whole mouse embryo) and Western (whole brain lysate) blotting we show that *LRRK2 *does not have an expression profile typical of a gene important during early development (Figure 4A,B). mRNA levels measured from whole mouse embryos show that *LRRK2 *expression increases from embryonic day 15 to day 17 (Figure 4A). Given the large increase in expression of LRRK2 during the latest stages of development, we created a panel of cDNA derived from developing mouse organs, including brain, lung, heart and liver, at various stages of development. We then went on to compare *LRRK1 *and *LRRK2 *levels using quantitative RT-PCR and observed a dynamic expression profile that distinguishes *LRRK1 *from *LRRK2*. *LRRK2 *most dramatically increases in lung and kidney between E15.5 and E19.5 partially accounting for the results of the whole-embryo Northern blot. Surprisingly, *LRRK2 *mRNA is constitutively expressed early in development of the brain and does not substantially increase upon cell maturation as might be expected by the Northern blot of whole embryo (Figure 4C). *LRRK1 *levels spike at E15.5 in the brain then decrease below LRRK2 levels and remain low with respect to *LRRK2 *throughout adulthood. In other organs such as the lung and liver, *LRRK2 *dramatically increases in the latest stages of development in contrast to the more constitutive expression in the heart where *LRRK1 *and *LRRK2 *are nearly identically expressed both in development and throughout adulthood.

## Discussion

The function of LRRK proteins in mammals remains enigmatic yet strong evolutionary conservation suggests important roles. Besides obvious interest in understanding the only proteins in the mammalian proteome encoding both a kinase and GTPase domain, the striking association between *LRRK2 *and PD further justifies the speedy discovery of LRRK2 protein function in cells. Within the present study we show that several commercially available antibodies recognize endogenous human LRRK2 protein but no tested commercially available antibody can efficiently resolve mouse LRRK2 protein despite a number of reports that did not have the benefit of *LRRK2*-null mice. To the contrary, more abundant cross-reactive species near or at the molecular weight of mLRRK2 protein are detected by most commercially available antibodies as shown for the human specific Novus antibodies and the Abgent antibody AP7099b. Cross-reactive species likely mask endogenous mLRRK2 protein in the case of antibodies from Cell Signaling and Chemicon. However, both antibodies are capable of detecting over-expressed mouse protein. The Alexis antibody detects over-expressed mouse protein and endogenous mouse protein that migrates slightly below cross-reactive species. It may be possible with additional purification steps, blocking optimization, extraction optimization and improved washing and blotting conditions that some of the antibodies tested here will prove useful in the future.

In all tissues, *LRRK1 *and *LRRK2 *mRNA is rare compared to even nominally expressed transcripts such as *TATA binding protein *(*TBP*) and *cyclophilin-A*, both used here in addition to *GAPDH *as normalization controls. The combination of a poorly expressed transcript with an apparently unstable protein of above-average size creates a worst case scenario for routine detection of endogenous protein by Western blotting and immunocytochemistry despite the high solubility of LRRK2 protein.

We describe cloning of mouse *LRRK2 *into expression vectors and the expression profile of *LRRK1 *and *LRRK2 *in the developing and adult mouse. Mouse LRRK2 expression increases from embryonic day 15 to day 17 and continues through adulthood. In some organs, such as the brain, *LRRK2 *expression apparently increases to a more nominal extent at earlier time points. We have been unable to detect LRRK2 protein in the brain before embryonic day 17 using the western blotting conditions and protein extraction protocols described here. *LRRK1 *and *LRRK2 *expression overlaps in all tissues used in this study (whole brain, eye, lung, skeletal muscle, smooth muscle, thymus, lymph node, heart, stomach, liver, kidney and gut), and in some tissues expression levels are identical. *LRRK2 *expression levels are particularly high in embryonic and adult lung and kidney. As kinase enzymes, LRRK protein may target tissue specific substrates for phosphorylation. It is possible that LRRK protein is so critical to cell function that the protein has diverged into two related proteins to provide a protective measure of redundancy in function.

## Conclusion

Knowledge about localization, function and substrates of LRRK1 and 2 protein will help clarify *LRRK2 *associated disease. We characterized twenty-one commercially available antibodies against LRRK2 protein and describe the biochemical properties of mouse and human LRRK2 proteins with regards to solubility and expression. The importance of *LRRK1 *and *LRRK2 *for mouse development and adult mice in different tissues is actively pursed by many research groups. *LRRK1 *and *LRRK2 *are comparably expressed in most tissues with stable expression in adulthood but dynamic expression during development, with a general trend towards increased expression during organogenesis and maturation.

## Methods

### Antibody production and western blotting

Affinity-purified rabbit polyclonal antibodies were generated to LRRK2 protein as described previously [17]. Protein extracts from adult mouse and fresh frozen tissue samples from the human temporal lobe were prepared as described [17] and Western blot analysis (6% SDS-PAGE gels) was conducted as previously described for *LRRK2 *[19]. Human tissue samples were obtained from human brain temporal lobe resections snap-frozen immediately after removal and stored at -80°C until homogenization. Briefly: whole mouse brain or temporal human brain was mechanically homogenized in either PBS (low salt buffer), high salt buffer (PBS supplemented to 600 mM NaCl), 1% Triton X-100 (in PBS), 1% SDS (in PBS) or RIPA (1% SDS, 1% sodium deoxycholate, 1% NP-40). All buffers contained complete protease inhibitors (Roche). Antibodies were diluted 1:1000 in TBS-T containing 5% skimmed milk if not otherwise indicated. Incubation with primary antibodies was performed overnight. *LRRK2 *knockout mice were generated by disruption of exons 39 and 40 of the mouse *LRRK2 *gene (S. Biskup, M. Sasaki, V.L. Dawson and T.M. Dawson, personal observation). Wild-type, heterozygote and knockout *LRRK2 *mice were generated by crossbreeding of germline chimaeras to C57BL/6J mice. Protein extracts from mouse and fresh frozen tissue samples from the human temporal lobe were processed for Western blotting with LRRK2 antibodies as described above. LRRK2 antibodies were obtained from Abgent, Novus Biologicals, Abcam, Chemicon, Alexis and Cell Signaling.

### Generation of human and mouse LRRK2 constructs

The generation of human *LRRK2 *constructs has been previously described [19,35].

The full-length cDNA of mouse *LRRK2 *was amplified from a Marathon-Ready mouse embryo 17d cDNA library (BD Biosciences). Using nested primers based on mouse *LRRK2 *(GenBank accession number NM_025730) and *PfuTurbo *hotstart DNA polymerase (Stratagene) according to the manufacturer's protocol, two overlapping fragments from exons 1–28 (~3.9 kb) and exons 15–51 (~5.9 kb) were generated. The first part of the *LRRK2 *cDNA (exons 1–28) was cloned in pBluescript SK+ (Stratagene) (*Kpn*I/*Not*I) and sequenced with an ABI Prism Big Dye Terminator cycle sequencing kit on an ABI Prism 3730 DNA analyzer (Applied Biosystems). To generate a full-length *LRRK2 *clone the second part of *LRRK2 *(exons 15–51) was cloned in the pBluescript plasmid already containing one part of *LRRK2 *(*Nde*I/*Not*I). The entire open reading frame of *LRRK2 *(7584 bp) was confirmed by sequencing.

### Northern blotting

Whole mouse embryonic membranes were purchased from Clontech. Membranes were hybridized overnight at 65°C with a biotinylated antisense riboprobe synthesized using the MAXIscript in vitro transcription kit (Ambion) from a PCR-amplified DNA template with incorporated T7 promoter, and washed with 2 × SSC/1% SDS and then 0.1 × SSC/0.1% SDS at 65°C. Filters were visualized by incubation with streptavidin-alkaline phosphatase and chemiluminescence using the BrightStar BioDetect kit (Ambion). The riboprobe sequence for *LRRK2 *corresponds to nucleotide positions 4292 to 4813 of *LRRK2 *cDNA. PCR primers used were: forward 5'-TGAAGCCTTGGCTCTTCAAT-3' and reverse 5'-TAATACGACTCACTATAGGTACAAAGCCACTTGGGTTCC-3' with bold nucleotides representing the T7 promoter priming site.

### Quantitative reverse-transcription PCR

RNA was isolated from embryonic and adult CD-1 mice (Charles Rivers) via mechanical homogenization and extraction in TriZOL and both cleaned and concentrated using a commercially available kit (Qiagen RNEasy Micro; Qiagen). Reverse transcription was performed using the Applied Biosystems High Capacity cDNA Reverse Transcription Kit (Applied Biosystems); cDNA concentrations were determined with a Nanodrop spectrophotometer (Nanodrop Technologies) prior to PCR. cDNA samples underwent absolute quantitative real-time PCR on the 9700HT instrument (Applied Biosystems) under the default 40-cycle program. TaqMan gene expression assays were purchased from Applied Biosystems, including *LRRK1 *and *LRRK2 *expression assays (assay Mm0481934 and Mm00713303), TBP (assay Mm00446973_m1), *cyclophilin A *(Mm02342429_g1) and *GAPDH *(4352932E). For each amplicon, PCR efficiency was estimated near 1.0 by serial dilutions of cDNA. Relative quantities of mRNA were estimated using the delta delta CT method.

## List of abbreviations

leucine-rich repeat kinase 2 (LRRK2), leucine-rich repeat kinase 1 (LRRK1), Parkinson's Disease (PD), polymerase chain reaction (PCR), human embryonic kidney cells (HEK cells), pheochromocytoma cells (PC12 cells), phosphate buffered saline (PBS), sodium dodecyle sulfate (SDS), SDS polyacrylamide gel electrophoresis (SDS-PAGE), formic acid (FA), real time PCR (RT-PCR), embryonic day (E), postnatal day (P), glyceraldehyde 3-phosphate dehydrogenase (GAPDH), TATA binding protein (TBP), sodium chloride/sodium citrate (SSC), Radioimmunoprecipitation (RIPA), sodium chloride (NaCl), Nonidet P-40 (NP-40), human temporal lobe (Htl), wild-type (WT), knock-out (KO).

## Authors' contributions

SB, ABW, AR and CC carried out all experiments, BLD generated the mouse *LRRK2 *clone, DJM developed and purified the JH5514 antibody. TMD and VLD provided reagent and infrastructure support for the experiments and critically reviewed the manuscript. All authors read and approved the final manuscript.
